# Editorial: Biomarkers in structural cardiovascular disease: insights into screening, diagnosis and prognosis

**DOI:** 10.3389/fcvm.2023.1337376

**Published:** 2023-11-23

**Authors:** Vitor Emer Egypto Rosa, Hector M. Garcia-Garcia, Carlos M. Campos, Roney Orismar Sampaio

**Affiliations:** ^1^ Instituto do Coração (InCor), Hospital das Clínicas, Faculdade de Medicina da Universidade de São Paulo (HC FMUSP), São Paulo, Brazil; ^2^ MedStar Washington Hospital Center, Washington, DC, United States; ^3^ Department of Hemodynamic, Instituto Prevent Senior, São Paulo, Brazil

**Keywords:** biomarkers, structural cardiovascular disease, diagnosis, prognosis, risk prediction, screening

**Editorial on the Research Topic** Biomarkers in structural cardiovascular disease: insights into screening, diagnosis and prognosis

Although the term “biomarkers” usually evokes the notion of a blood test, by definition, biomarkers encompass any objectively measurable characteristics, be they molecular, histological, radiographic, or physiological (1). Their utilization is on the rise in medical practice, serving as promising indicators of normal biological processes, disease risk prediction, diagnosis, prognosis, and monitoring response to treatment (1). Consequently, biomarkers hold the potential to address gaps in our understanding of highly prevalent cardiac pathologies, especially in structural heart disease, which remains the leading cause of death globally and also leads to the loss of physical function, quality of life, and excess health system costs (2, 3). The present Frontiers Research Topic gathers studies regarding biomarker assessment in structural cardiovascular disease in an attempt to enrich medical literature on this crucial topic.

Patent foramen ovale (PFO) is a structural abnormality with high prevalence in the general population. The most concerning complication is cryptogenic stroke. However, PFO closure is only indicated after an embolic stroke (4). Thus, Liu et al. evaluated predictors of stroke in the PFO population, the change in PFO height during the Valsalva, and a simple echocardiographic score comprising four items that can help identify patients at higher risk of stroke, contributing to selecting patients who will benefit from PFO intervention.

High-sensitivity troponins are the gold standard for the diagnosis of non-ST-elevation myocardial infarction, and their application is progressively extending to various scenarios. An illustrative example is found in the comprehensive literature review conducted by Leite et al., which explores all aspects relating to the use of cardiac troponins as risk markers of future cardiovascular events. Although additional research is needed, troponin appears to be a potential tool for risk prediction, as summarized by the authors.

Another aspect related to the use of serum biomarkers involves the assessment of myocardial injury. Definitions can vary based on cardiology societies and clinical scenarios. Even when evaluating the same pathology, such as aortic stenosis, definitions may vary depending on the type of intervention. Specifically, in the context of transcatheter aortic valve intervention (TAVI), the impact of periprocedural myocardial injury is uncertain. Therefore, de Sá Marchi et al. conducted a meta-analysis to investigate the influence of CK-MB, troponin, and VARC-2 definition on mortality following transcatheter aortic valve replacement (TAVR), as well as the specific period during which myocardial injury has a major impact.

Acute pulmonary embolism (APE) is a life-threatening condition with a broad spectrum of presentations. There is an increasing need to better understand APE pathophysiology, with the aim of enhancing diagnosis and preventive care. Consequently, the metabolomic approach emerges as a compelling and innovative biomarker that can be integrated into these strategies. To address these questions, Xie et al. evaluated the variations in metabolic profile among patients with APE, non-ST-segment elevation myocardial infarction, and a healthy group. This assessment was conducted using ultra-performance liquid chromatography-mass spectrometry, employing an untargeted metabolomics approach.

COVID-19 is also associated with pulmonary thrombosis. However, it is noteworthy that patients without APE in computed tomography (CT) scans may still present respiratory failure. Post-mortem studies suggest that obstructive disease in the microvascular lung vessels may be one of the underlying mechanisms. This is the basis of the study conducted by Hajjar et al., who performed optical coherence tomography in patients with APE diagnosed in CT scans, as well as those with negative CT scans but elevated thromboinflammatory markers.

Finally, cardiac biomarkers have a crucial role in the management of patients with valvular heart disease, particularly in cases of aortic stenosis. B-type natriuretic peptide (BNP) and N-terminal pro-BNP (NT-pro BNP) exhibit distinct behavior in patients with aortic stenosis compared to those with heart failure. Nonetheless, they still may have an impact on diagnosis, progression assessment, intervention indication, and prognostication of aortic stenosis. Cavalcante et al. provide a concise overview of the utility of cardiac biomarkers in the context of aortic stenosis, highlighting both their limitations and strengths across various clinical scenarios.

Low-flow, low-gradient aortic stenosis with low ejection fraction represents a high-risk entity for both transcatheter and surgical aortic valve replacement. The progressive deterioration of imaging parameters of bi-ventricular remodeling and left ventricle fibrosis may hold a significant prognostic impact. Therefore, identifying simple methods for this evaluation may prove beneficial for clinical practice. Thus, Lopes et al. examined the relationship between the combination of BNP and high-sensitivity troponin I with multimodality imaging methods. These included T1 mapping cardiac magnetic resonance, echocardiogram, and dobutamine stress echocardiography.

NT-pro BNP may also have a significant impact on the long-term prognosis of patients with severe aortic stenosis, including those undergoing TAVI. Zhou et al. investigated the influence of baseline NT-pro BNP levels on mortality 5 years post-TAVI. Additionally, the authors also assessed the effect of changes in NT-pro BNP levels at 30 days on the same outcomes.

This Research Topic regarding biomarkers in structural heart disease brings provocative new concepts that can support further research in this area. As an evolving field, there is a pressing need for additional research to refine the application of these tests in clinical practice and enhance our comprehension of the underlying pathophysiology of various structural diseases. This compilation may help stimulate more studies in this critical area.

## References

[1] Paczesny S Hakim FT Pidala J Cooke KR Lathrop J Griffith LM National institutes of health consensus development project on criteria for clinical trials in chronic graft-versus-host disease: III. The 2014 biomarker working group report. Biol Blood Marrow Transplant. (2015) 21(5):780–92. 10.1016/j.bbmt.2015.01.003 25644957 PMC4408233

[2] Mensah GA Roth GA Fuster V. The global burden of cardiovascular diseases and risk factors: 2020 and beyond. J Am Coll Cardiol. (2019) 74:2529–32. 10.1016/j.jacc.2019.10.009 31727292

[3] Roth GA Mensah GA Fuster V. The global burden of cardiovascular diseases and risks: a compass for global action. J Am Coll Cardiol. (2020) 76:2980–1. 10.1016/j.jacc.2020.11.021 33309174

[4] Mas JL Derumeaux G Guillon B Massardier E Hosseini H Mechtouff L Patent foramen ovale closure or anticoagulation vs. antiplatelets after. Stroke N Engl J Med. (2017) 377:1011–21. 10.1056/NEJMoa1705915 28902593

